# Keratin K15 as a Biomarker of Epidermal Stem Cells

**DOI:** 10.3390/ijms141019385

**Published:** 2013-09-25

**Authors:** Amrita Bose, Muy-Teck Teh, Ian C. Mackenzie, Ahmad Waseem

**Affiliations:** Centre for Clinical and Diagnostic Oral Sciences, Barts and the London School of Medicine and Dentistry, Queen Mary University of London, Turner Street, London E1 2AD, UK; E-Mails: amrita.bose@savingfaces.co.uk (A.B.); m.t.teh@qmul.ac.uk (M.-T.T.); i.c.mackenzie@qmul.ac.uk (I.C.M.)

**Keywords:** transit-amplifying cells, keratinocyte differentiation, basal keratinocytes, intermediate filaments, pluripotent cells

## Abstract

Keratin 15 (K15) is type I keratin protein co-expressed with the K5/K14 pair present in the basal keratinocytes of all stratified epithelia. Although it is a minor component of the cytoskeleton with a variable expression pattern, nonetheless its expression has been reported as a stem cell marker in the bulge of hair follicles. Conversely, suprabasal expression of K15 has also been reported in both normal and diseased tissues, which is inconsistent with its role as a stem cell marker. Our recently published work has given evidence of the molecular pathways that seem to control the expression of K15 in undifferentiated and differentiated cells. In this article, we have critically reviewed the published work to establish the reliability of K15 as an epidermal stem cell marker.

## 1. Introduction

### What Are Stem Cells?

The hierarchy of embryonic stem cells begins with the totipotent zygote and extends up to the morula stage of the embryo, in which all cells are capable of generating an entire organism. This is followed by the pluripotent blastocyst stage, where cells of the inner cell mass have the capacity to form ectoderm, mesoderm and endoderm and the germ cells. However, in adult tissues, the stem cells become only multipotent, although remaining capable of undergoing unlimited self-renewal to provide a source of cells for their surrounding tissues. This enables the organism to repair and/or regenerate lost tissue and is critical for survival [1].

Two distinct features differentiate a stem cell from a non-stem cell population. First, stem cells possess an unlimited capacity of self-renewal, which enables them to undergo continuous cell division throughout the life of an organism. Second, stem cells are capable of undergoing asymmetric cell division to produce two daughter cells with different differentiation capacities. While one enters an irreversible differentiation pathway, the other is retained *in situ* with an unlimited capacity to divide and thus form the next generation of stem cells. The cell committing to differentiation is termed a “transit-amplifying” (TA) or “progenitor” cell, characterised by finite self-renewal capacity and commitment to terminal differentiation following a few cell divisions. This hierarchy is unidirectional and TA cells do not normally regain stem cell characteristics [2]. Although the presence of ordered columnar structure and “epidermal proliferative units” has been a widely accepted model for adult epidermal homeostasis, studies with transgenic mice failed to detect such a pattern and suggested an alternative pattern in which stem cells form a small quiescent population that is activated only for regeneration after injury; renewal for epidermal homeostasis is due to a single progenitor population undergoing random, but balanced, symmetric and asymmetric cell division patterns [3]. More recently, however, it has been shown that the promoter used to drive marker expression is of particular importance to experimental studies of epithelia in transgenic mice and that use of a K14 promoter enables a stem and amplifying pattern to be demonstrated [4]. Studies with similar transgenic mice, also using a marker driven by K14, indicate that the stem cell population in oral mucosa forms the cells of origin for oral carcinomas [5].

Within a tissue, stem cells reside within a defined area called the “niche”, which consists of supporting cells and extracellular factors that are conducive to maintaining stem cell characteristics. For example, in the human skin, such a niche was first detected in the bulge of hair follicles by using C8/144B monoclonal antibody. This antibody preferentially immunostained the bulge keratinocytes without staining other parts of the hair follicle and the protein it cross-reacted with was found to be K15. This was one of the first studies to correlate K15 expression with epidermal stem cells [6]. It was also demonstrated that the bulge cells, compared to TA cells, retained DNA precursor label, bromodeoxyuridine, thereby signifying that they underwent limited cell division [7]. The bulge cells have been shown to generate all the different epithelial cell lineages, such as both follicular and interfollicular keratinocytes, sebaceous gland cells and the hair matrix cells and are referred to as “follicular stem cells” [8,9]. Although several studies have reported the importance of the follicular stem cells in epidermal repair and maintenance, however, presence of discrete epidermal proliferative units, consisting of stem and committed TA cells, has recently been identified in the interfollicular epidermis. These studies have reported that the slow-cycling stem cells located in the interfollicular compartment are primarily responsible for repair and regeneration of the non-follicular epidermis [4].

## 2. Evolution of Epidermal Stem Cell Research

The expansion of epidermal stem cell research from the 1960s until the present has been tremendous. It started with the recognition of units of epidermal structure in which the suprabasal cells and the superficial corneocytes are stacked to form cell columns [10] and then demonstration of proliferative heterogeneity amongst the basal cells beneath such units [11]. Thereafter, a hierarchy of proliferative epithelial cells, containing a small number of basal stem cells was suggested [12,13]. During the 1980s–1990s, several authors used label retaining assays and clonal analysis to identify and establish the hierarchy produced by epidermal stem cells and, finally, defined the bulge of the hair follicles as the site of epidermal stem cells in humans [14–16]. Advanced molecular biology techniques during the last decade have given evidence of interfollicular stem cells using lineage tracing *in vivo*, have detected the immigration of bone marrow-derived stem cells to the epidermis during epidermal regeneration, have identified the importance of p63 in stem cell differentiation and initiation of epithelial stratification, and have derived keratinocytes from induced pluripotent cells and *vice-versa* [17].

### Epidermal Stem Cell Markers

The etio-pathogenesis of cutaneous neoplasms has been attributed to the resident epidermal stem cells, as the long life span of these cells makes them a likely target for oncogenic mutations [18]. Hence, it has become an absolute necessity to identify and characterise these cells to gain insight into epidermal cancer formation, progression and metastasis. Identification of differences between stem cells and the TA cell population also enable a better understanding of the dynamics of keratinocyte biology.

Although, substantial research has been done to identify epidermal stem cells, the reliability of stem cell markers still remains debatable. Several variables, such as species variance between mouse and humans, type of body tissue, presence or absence of hair, *in vivo* and *in vitro* conditions and the isolation techniques employed to identify the stem cells, have been discussed as the primary reason for the ambiguity of markers [19]. Of the several epidermal stem cell markers identified, the expression of β1 integrins has been widely accepted. It has been demonstrated that those human keratinocytes, both cultured and derived from *in vivo* foreskin, which adhere most rapidly to type IV collagen or fibronectin, also express the highest level of β1 integrin on their surface. These cells were found to divide actively and form larger colonies, a characteristic typical of stem cells in culture [20]. Another study has reported that stem cells derived from murine or neonatal human foreskin, express high levels of α6 integrin and low levels of CD71 transferrin receptor. Such cells were found to exhibit a small blast-like phenotype with high self-renewal capacity and low expression levels of K10, a keratin expressed in the differentiated suprabasal layers of stratified epithelia [21]. Epidermal stem cells have also been found to express markers that are typically expressed by stem cells in other tissues. For example, murine bulge keratinocytes have been found to express CD34, a haematopoietic stem cell marker, and these CD34+ cells formed large colonies, were slowly cycling with a high label-retaining capacity, existed in the G0/G1 phase of the cell cycle, expressed high K15 levels, and stained intensely for α6 integrin [22]. An intestinal stem cell marker, Lgr5, has also been used to identify multipotent stem cells of the mouse hair follicle capable of giving rise to new hair follicles with maintenance of all cell lineages [23]. Recently cells containing Lgr6, a closely related molecule to Lgr5, have been proposed to be the markers of the most primitive stem cells present in the epidermis [24]. Cell surface biomarkers have formed the traditional methods for distinguishing stem cells from the TA and terminally differentiating keratinocytes. However, a recent study has demonstrated that the innate biochemical composition of a keratinocytes can be used for the same purpose and that infrared spectroscopy can be used to detect the vibrational modes of phosphate molecules, PO_2_^−^, present within the keratinocytes. Such vibration modes were found to be different between the stem, TA, and terminally differentiated cells, with each cell type expressing a distinct DNA conformation [25].

## 3. K15—A Marker of Stem Cells

Several authors have described K15 as a putative epidermal stem cell marker. One of the first studies to correlate K15 expression with human epidermal stem cells reported (a) that a cross reacting monoclonal antibody C8/144B preferentially stained keratinocytes of the hair follicle bulge and (b) that the protein it cross-reacted with was K15 [6]. These bulge cells demonstrated stem cell features and were slowly-cycling, had high levels of β1 integrin expression, preferentially proliferated during anagen phase of hair cycle, which reflects the growth phase wherein the stem cells rapidly divide, and also expressed K19, a reported cutaneous stem cell marker. On the basis of these observations it was suggested that both K15 and K19 could be used as markers of stem cells to segregate them from the differentiated progenitor population. The same study also reported that between the two keratins, K19 was found to be expressed more in the TA cells that had left the stem cell compartment, characterised by β1 integrin dullness, while those keratinocytes that were K15+ represented a more undifferentiated state, marked by β1 integrin brightness and label retaining capacity [6]. High expression of K19 has also been reported to co-localize with increased brightness of α3β1 integrin, a marker of slow cycling keratinocytes of the bulge. However, the authors also observed that K19 could not label any of those cells of the interfollicular region that retained [^3^H]thymidine, thereby questioning the reliability of using K19 as a marker of all cutaneous stem cells [26].

The observation of Lyle and co-workers [6] has been further suported by several other studies including specific localisation of K15 in stem cells residing in the bulge [27], preferentially targetting hair follicle bulge cells by mouse K15 promoter in adult K15/lacZ transgenic mice [28], and reconstruction of all components of skin epithelium by K15+ cells [29]. Furthermore, expression of K15 mRNA and protein has been detected in the human anagen bulge which represents the onset of a new hair follicle growth phase [30]. K15+ cells in the mouse epidermal bulge were shown to coincide with Lgr5+ cells, which were able to regenerate new hair follicles and maintain all cell lineages of the follicle [23]. K15+ bulge cells from human skin also stained postively for another epidermal stem cell biomarker, CD200, and such cells had a much higher colony-forming ability [8]. A recent study of scalp of patients suffering from androgenetic alopecia, showed that K15 postive cells were smaller in size and had a much lower rate of proliferation, suggestive of a stem cell phenotype [31].

### 3.1. Functions of Keratin K15

The mitotically active basal layer of stratified epithelia expresses the major keratin pair of K5 and K14 and a minor type I keratin K15, which lacks a natural co-expression partner [27,32–34]. In normal stratified epithelia, the expression of K5/K14 and K15 is confined to the proliferating basal keratinocytes and ceases to express when these cells commit to terminal differentiation and begin their journey towards the surface. This journey is characterised by the expression of K1 and K10 in the suprabasal layer and K2 in the upper spinous layers. Such changes demonstrate the differentiation-specific expression of keratins [33]. While the expression of K5 and K14 is present uniformly in all the basal keratinocytes of all stratified epithelia, the expression of K15 differs in different tissues. For example, in the epidermis, expression of K15, although restricted to the basal layer, is present in patches. The cells at the deep rete ridges show strong expression of K15, whereas those overlying dermal papillae show little or no expression [21,27,34,35]. However, in internal epithelia, such as the oral mucosa, K15 expression is uninterrupted [36]. In the neonatal human skin and very young epidermis (~1.5 years), its expression is present throughout the basal layer [37], which may indicate a role for K15 in the developing epidermis. To our knowledge, K15 is the only basal-specific keratin that has been reported to have a suprabasal expression in normal esophagus [27,34,38] and in pathological conditions, such as oral lichen planus [39].

Expression studies on developing human embryos have shown that K15 is one of the earliest stratification-related keratins to be expressed in all types of developing stratified epithelia and, together with K4. K15, continues to be expressed in adult epidermis as a minor component [38]. As K15 is one of the basal-specific cytoskeletal proteins, providing structural support to the basal layer may be one of its primary functions but the discontinuous nature of K15 expression in the adult epidermis indicates that its presence maybe essential for the structural integrity of the basal layer only in the developing epidermis and not in adults. Therefore, K15 must have another function in the adult epidermis that is compatible with its selective expression pattern but, as yet, the K15 function in adult stratified epithelia is not understood.

That the main function of K5 and K14 is to provide structural support to the basal layer of stratified epithelia is evident by the fact that mutations in either of this pair leads to the pathogenesis of an inherited group of blistering disorder, collectively termed epidermolysis bullosa (EB) [40]. Most of the causative mutations are missense in nature and they are inherited as dominant negative [41]. However, recessive missense mutations in K5 and K14 have also been reported [41]. In some cases homozygous nonsense mutations in K14 lead to premature termination and the patients are completely devoid of K14 protein. These EBS cases are extremely rare and are recessive in nature [42]. EBS patients completely devoid of K5 have not been reported in humans and this suggests that absence of K5 may be embryonic lethal. The absence of K14 could be compensated by K15 but as K5 is the only type II keratin present in the basal layer, its absence cannot be compensated by another keratin. Absence of K5 in the basal layer would remove all type I keratins by ubiquitinylation and, making the basal keratinocytes devoid of keratin filaments, would consequently destabilise the cyto-architecture of the basal layer [43]. No mutation in keratin K15 has ever been associated with EBS, or any other blistering diseases, suggesting either that K15 mutations do not exist, either because they are embryonic lethal or perhaps they are yet to be discovered. Given the pattern of K15 expression during embryonic development, together with a possible stem cell function, it is plausible that embryos harbouring a K15 mutation would not survive.

To compensate for loss of K14 function, K15 expression would need be elevated in EBS patients where K14 is completely absent. While some studies have provided evidence for a compensatory role of K15 [32,44], others have reported no such compensation [42]. In 1995, Lloyd and co-workers observed that K14 knockout mice had a relatively unaffected oesophagus but had blistered skin. This was attributed to high level of K15 expression in the oesophagus [32]. This led to the hypothesis that K15, if expressed at high levels, will be able to compensate for the absence of K14. Up-regulation of K15 expression in the epidermis of 4 EBS patients with ablated K14 has been reported and in these patients K5 was able to polymerise with K15 but the K5/K15 pair could form only 6 nm wispy proto-filaments instead of mature 10 nm keratin filaments [44]. In an independent study, a similar upregulation of K15 expression in the epidermis of a patient suffering from natural ablation of both K14 alleles has also been reported [27]. However, a study of a patient suffering with Köbner form of EBS, characterized by complete absence of K14 in the epidermis, has reported no compensatory increase of K15 in the basal cells to form filaments with K5 [42]. Therefore, at present it is uncertain whether K15 can functionally compensate for the absence of K14 in EBS patients. This aspect is worthy of further investigation.

As stem cells continue to divide throughout the life of an organism, such cells are at a high risk of accumulating genetic mutations and could be potential targets of tumour initiation [45,46]. For example, the bulge stem cells have been implicated in the carcinogenesis of trichoepitheliomas (TE) and basal cell carcinomas (BCC), as both lesions have a histological similarity with the bulge cells [9]. K15 expression in TEs is much higher at the periphery of the lesion than for BCCs. It was therefore possible to use K15 expression to distinguish between the two lesions [47]. This is of particular importance as TE is a benign lesion while BCC is a malignant skin neoplasm, so a correct diagnosis is essential to form the best treatment plan. In sebaceous tumours a subpopulation of cells which were less differentiated than other cells, suggesting a stem cell phenotype, expressed high levels of K15 [45].

The study of K15 expression in the two most common epithelial malignancies, BCC and squamous cell carcinomas (SCC), highlights a role of K15 in influencing the behaviour of these lesions. While BCC is a locally invasive epidermal malignancy with highest rate of incidence, SCC is the second most common epithelial malignancy and has a significant propensity to metastasise [18]. Several studies have reported that BCCs express high levels of K15, whereas SCCs have been reported to express very low levels [9,18,34]. This expression pattern may indicate a role for K15 in maintaining the epithelial lineage of keratinocytes in BCC, thereby inhibiting metastasis. Downregulation of K15 in SCC may have a role in inducing the mesenchymal changes in keratinocytes that make them motile and therefore metastatic. This hypothesis is further supported by studies reporting downregulation of K15 in keratinocytes during wound healing [27,48]. Furthermore, during epithelial-mesenchymal transition (EMT), a developmental process leading to increased cell motility, there is a significant reduction in expression of cytokeratins, including K15. A role for EMT has also been implied during oncogenesis, and especially related to local invasion and metastasis [49,50].

When comparing keratin expression profiles of SCC samples from tongue, gingiva, floor of the mouth, buccal mucosa and palate with tissues obtained from similar regions of normal volunteers, it was found that while K14 is upregulated in oral SCC, K15 expression was significantly downregulated and was almost absent in dysplastic oral tissues [51]. Another study by Troy and co-workers also reported that K15 expression was progressively downregulated and ultimately lost in an epidermal tumorigenesis model that was generated by two stage chemical treatments with DMBA and TPA. However, in this model K14 was found to be expressed throughout the basal and suprabasal layers as well as tumour islands infiltrating the underlying dermis [52]. These studies not only emphasise the importance of K15 expression in diagnosis of epidermal tumours, but also highlight that dysplastic changes in SCC keratinocytes suppress K15 and induce K14. This perhaps suggests that K14 and K15, the two type I basal keratins, are either regulated by independent mechanisms or by a common mechanism that has an inverse effect on their expression.

### 3.2. K15 Is a Target of Stem Cell Specific Transcription Factor FOXM1

The human Forkhead Box M1 (FOXM1) protein belongs to a winged-helix transcription factor family of at least 50 unique *FOX* genes identified in the human genome [53]. Transgenic and knockout mouse studies have provided valuable information and confirm a pivotal role for FOXM1 in cell cycle regulation, cell-fate determination, embryonic development, adult tissue homeostasis, organ regeneration and ageing (reviewed in [54,55]. Emerging evidence has indicated that FOXM1 plays an important role in maintaining stem cell renewal through pluripotency genes *Oct4*, *Nanog* and *Sox2* in mouse [56–58]. A recent mouse model study established a key role for FOXM1 in cell fate determination and showed that FOXM1 regulated mammary luminal cell fate by modulating the expression of GATA-3, a key regulator of breast luminal epithelial differentiation [59].

Given a role of K15 as a stem cell marker, it is not surprising that a stem cell-related transcription factor such as FOXM1 may be an upstream target of K15. Indeed, K15 has been shown to be activated dose-dependently by FOXM1 in primary normal human keratinocytes whereby ectopic expression of FOXM1 was found to induce stem cell expansion and produce a hyperplastic phenotype in an organotypical culture system [60]. A FOXM1 DNA-binding motif is present within the promoter region of *K15* gene and it has been demonstrated, using the chromatin immunoprecipitation method, that FOXM1 protein indeed binds to the DNA-binding motif within the promoter of *K15* gene in human keratinocytes [61]. This finding is in agreement with the fact that FOXM1 and K15 are co-expressed in the rete-ridges of epidermis [21,60] and in the outer root sheath including the bulge, a putative stem cell compartment, of the hair follicle [62]. Furthermore, both K15 and FOXM1 are upregulated in basal cell carcinomas [9,34,63].

### 3.3. Is K15 a Reliable Stem Cell Marker?

In spite of substantial evidence in the literature supporting K15 to be a stem cell marker, several authors have questioned the reliability of correlating its expression with stem cell properties of keratinocytes. For example, in sheep hair follicles, K15 has been shown to be expressed in the outer root sheath and absent in the bulge region that is thought to contain the stem cells [64]. Contrary to the previous studies, a continuous K15 expression in the outer root sheath of human hair follicles, basal layer of epidermis and in eccrine glands has been reported recently [65]. Furthermore, in the internal oral and vaginal epithelia, uninterrupted K15 expression throughout the basal layer has been reported [36] and it is highly unlikely that every K15+ cell is a stem cell. Porter and co-workers have suggested K15 to be a marker of laterally differentiating epidermal keratinocytes in the basal layer [34]. Other recent studies have suggested that high K15 expression may not always mark a stem cell subpopulation and that basal keratinocytes expressing high levels of K15 are possibly those undergoing an abnormal differentiation programme [52]. Furthermore, K15 is reported to be expressed suprabasally in differentiating keratinocytes of normal human esophagus [32,38] and in oral lichen planus [39], and in freshly-cut skin sections exposed to thyroid hormone or IFNγ [66], findings inconsistent with an undifferentiated keratinocyte phenotype, a distinctive feature of stem cells. *In vitro* studies on keratinocyte differentiation have reported K15 expression only upon reaching confluence [34,67], which again is a characteristic feature of differentiation-specific genes. The expression of K15 has also been reported in non-epithelial tissues, such as the lymphoid tissue, which again questions the reliability of its expression exclusive to epidermal stem cells [68]. These studies taken together would question the status of K15 as a genuine “stem cell marker”.

**Figure 1:**
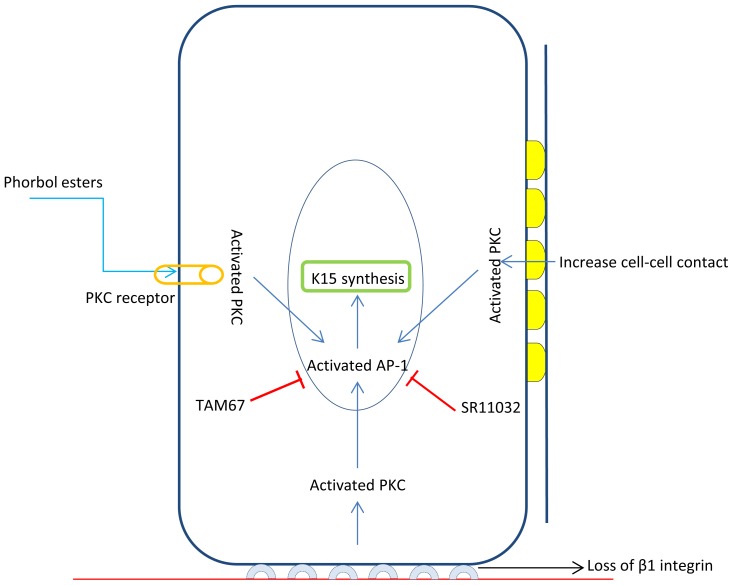
Schematic representation of the molecular signalling responsible for the differentiation-specific induction of Keratin 15 (K15) expression. Based on our previously published data we propose that K15, although normally a basal-specific keratin, can be induced in differentiating keratinocytes via Protein Kinase-C (PKC)/AP-1 pathway. Differentiation-specific signals, such as loss of cell-surface β1 integrin receptors, increased cell-cell interactions or exposure to tumour-promoter phorbol esters, activate the endogenous PKC signalling, which results in activation of downstream transcription factors AP-1, which in turn induce *K15* gene transcription. Blocking the activation of AP-1 by specific inhibitor, SR11302, or by dominant negative form of c-Jun, TAM67, inhibits the transcriptional induction of K15 [61].

In our recently published work, we have shown that differentiating keratinocytes are also capable of expressing K15. *In vitro* experiments conducted to trigger keratinocyte differentiation, expression of K15 could be induced through loss of cell surface β1-integrin receptors in suspension culture, increased cell-cell interaction in confluent monolayer cultures, or by direct exposure to differentiation-inducing chemical, such as phorbol 12-myristate 13-acetate (PMA) (Figure 1). We reported for the first time that such differentiation-specific induction is mediated by the Protein Kinase-C pathway (PKC) via AP-1 transcription factor which was capable of triggering expression of K15 along with other differentiation-specific biomarkers, such as K1, K10, involucrin and cornifin while downregulating the expression of the other type I basal keratin, K14 and FOXM1B, a cell cycle regulated transcription factor [61]. Our results further question the reliability of using K15 on its own as a biomarker for identifying any particular keratinocyte phenotype, and suggest that keratinocytes are capable of switching the expression of K15 in various ways under different conditions.

## 4. Conclusions

The proliferating basal layer of normal epidermis contains stem cells, TA cells and some keratinocytes committed to the differentiation pathway and in the process of migrating upwards. Expression of K15 in the basal layer has been reported a marker of stem cells and is still being used to distinguish stem cells from the TA and committed keratinocyte populations. However, several reports have questioned the reliability of K15 for this purpose. We have scanned the literature for studies relevant to the expression of K15 as an epidermal stem cell marker and found some reports supporting, as well as some refuting, a relationship. Although a complete explanation will require further investigations, through our recently published work on K15, we have provided an explanation for this contradiction. We propose that two putative mechanisms regulate K15 expression in keratinocytes: one that drives its expression in the basal layer, mediated primarily by FOXM1, and another that induces its expression in the suprabasal layers, involving PKC/AP-1 signalling [61]. The over all conclusion of the review is to highlight that K15 could be expressed in the stem cells as well as in differentiated cells. Therefore, use of this marker on its own may not provide conclusive information about the stem cell population in a tissue.
